# Supplementary material for: Enhanced Electrophoretic Depletion of Sodium Dodecyl Sulfate with Methanol for Membrane Proteome Analysis by Mass Spectrometry
Source: Proteomes. 2024 Feb 2;12(1):5. doi: 10.3390/proteomes12010005 (PMC10885059; doi:10.3390/proteomes12010005)
Supplement: Supplementary file 1 [file proteomes-12-00005-s001.zip › Supplemental_revised_jan26.pdf]

Supporting Information for

Enhanced Electrophoretic Depletion of Sodium Dodecyl Sulfate with Methanol for Membrane Proteome Analysis by Mass Spectrometry

Hammam H. Said and Alan A. Doucette *

Supplementary Materials:

Figure S1: Flow scheme summarizing membrane proteome solubilization experiment with methanol

Figure S2: Transmembrane electrophoresis device

Figure S3: SDS PAGE image of proteins collected following SDS depletion by TME in water or in 40% methanol

Figure S4: Plot of conductivity vs SDS concentration indicating the CMC of SDS in water, as well as 10, 20 and 40% methanol

Figure S5: Histogram of the number of transmembrane segments for proteins enriched in methanol vs water

Figure S6: Histogram of isoelectric points for proteins enriched in 40% methanol vs water

Also available:

Table S1: List of proteins identified along with their relative abundance;

Table S2: Gene Ontology Data for proteins enriched in 40% methanol and in water.

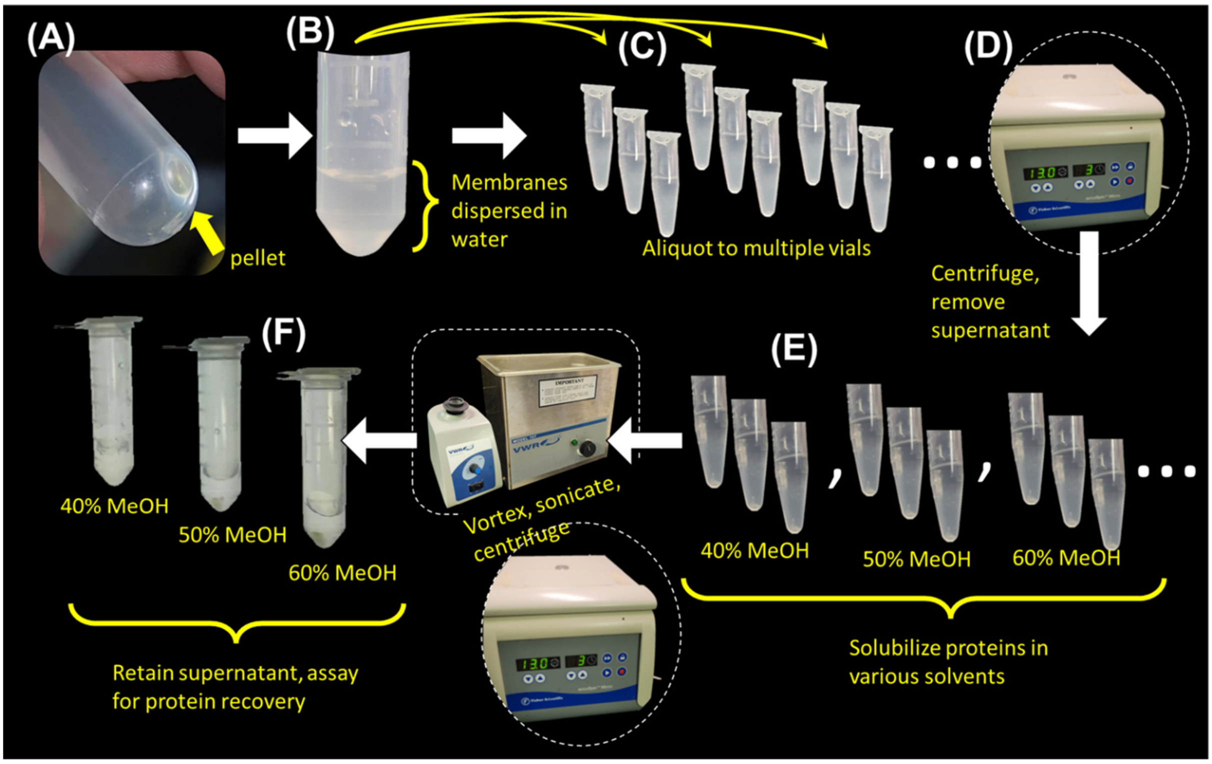

Figure S1. Flow chart summarizing membrane proteome extraction in methanol/water. (**A**) An enriched membrane fraction was prepared by ultracentrifugation of lysed yeast cells; (**B**) the pellet was agitated to form a homogeneous suspension of undissolved membranes in water; (**C**) Equal volumes of the suspension were aliquoted to multiple vials, thus transferring an equivalent quantity of membranes to test protein solubilization efficiency; (**D**) the suspension was centrifuged, and the supernatant discarded, resulting in the final pellet to test proteome solubilization efficiency; (**E**) The appropriate solvent system (ie 0.5% SDS in water, or varying ratios of methanol/ water) were added to the vials, testing three replicates per condition; (**F**) Following vortex and sonication, solubilized proteins were collected following centrifugation to remove the undissolved proteins in the membrane pellet.

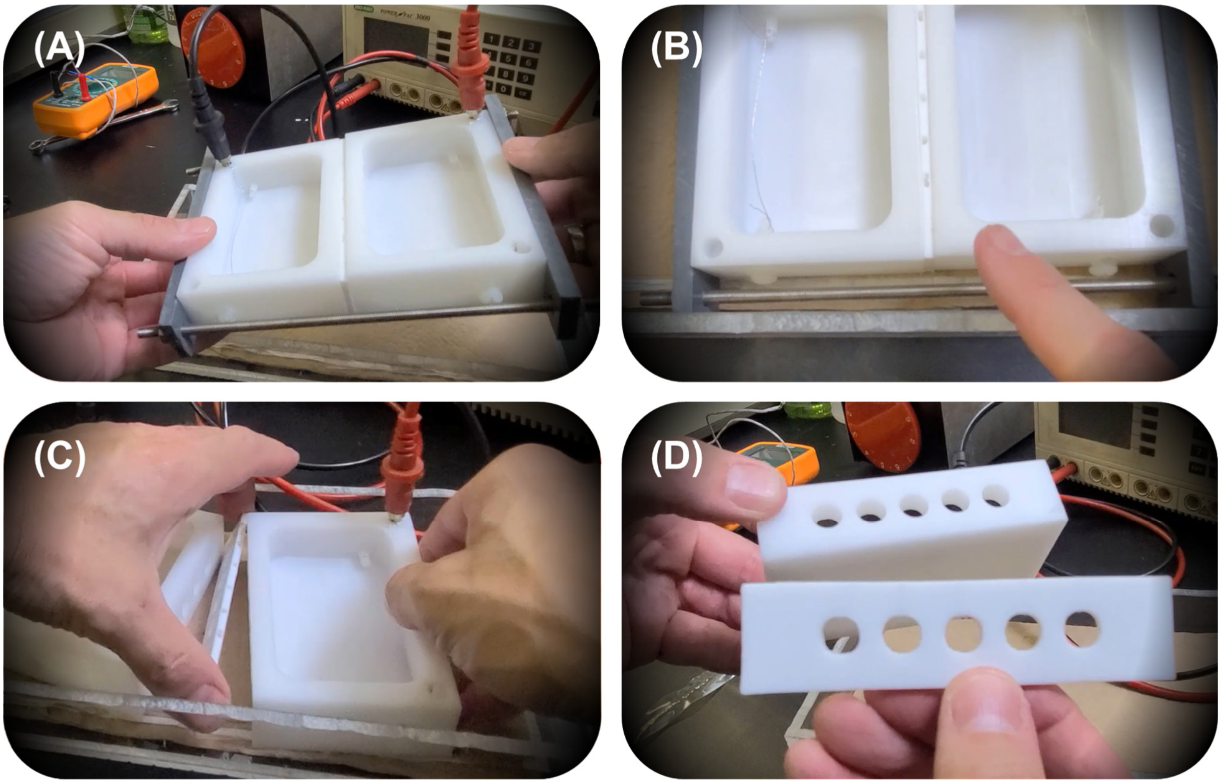

Figure S2. Transmembrane electrophoresis system (**A**) Shown is the fully assembled system, prior to depositing buffer; (**B**) samples are deposited into holes of the central sample plate, sandwiched by dialysis membranes, with the cathode and anode buffers to either side; (**C**) the sample cell is only 3 mm thick; (**D**) holes are machined into the central plate to form three sample cells, each capable of retaining up to 250 µL of sample.

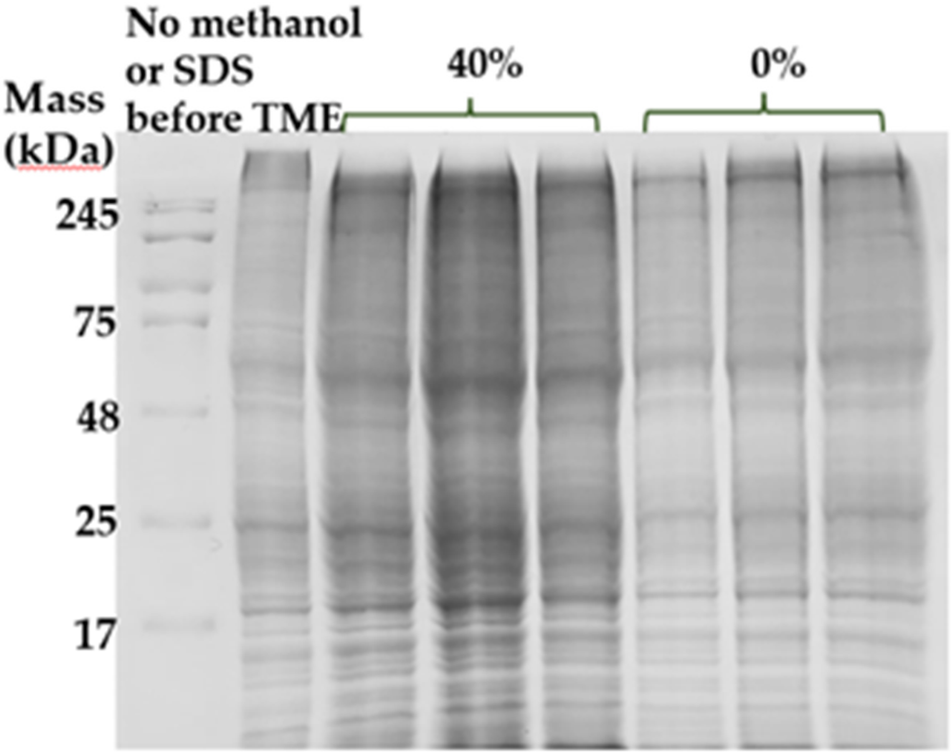

Figure S3. SDS PAGE of membrane proteome fractions initially extracted with 0.5% SDS and depleted of the surfactant using TME. It required 9 minutes for SDS to be reduced below 100 pm in the water preparation, while only 3 minutes for the sample containing 40% methanol. Shown are the proteins recovered in the soluble fraction following TME depletion.

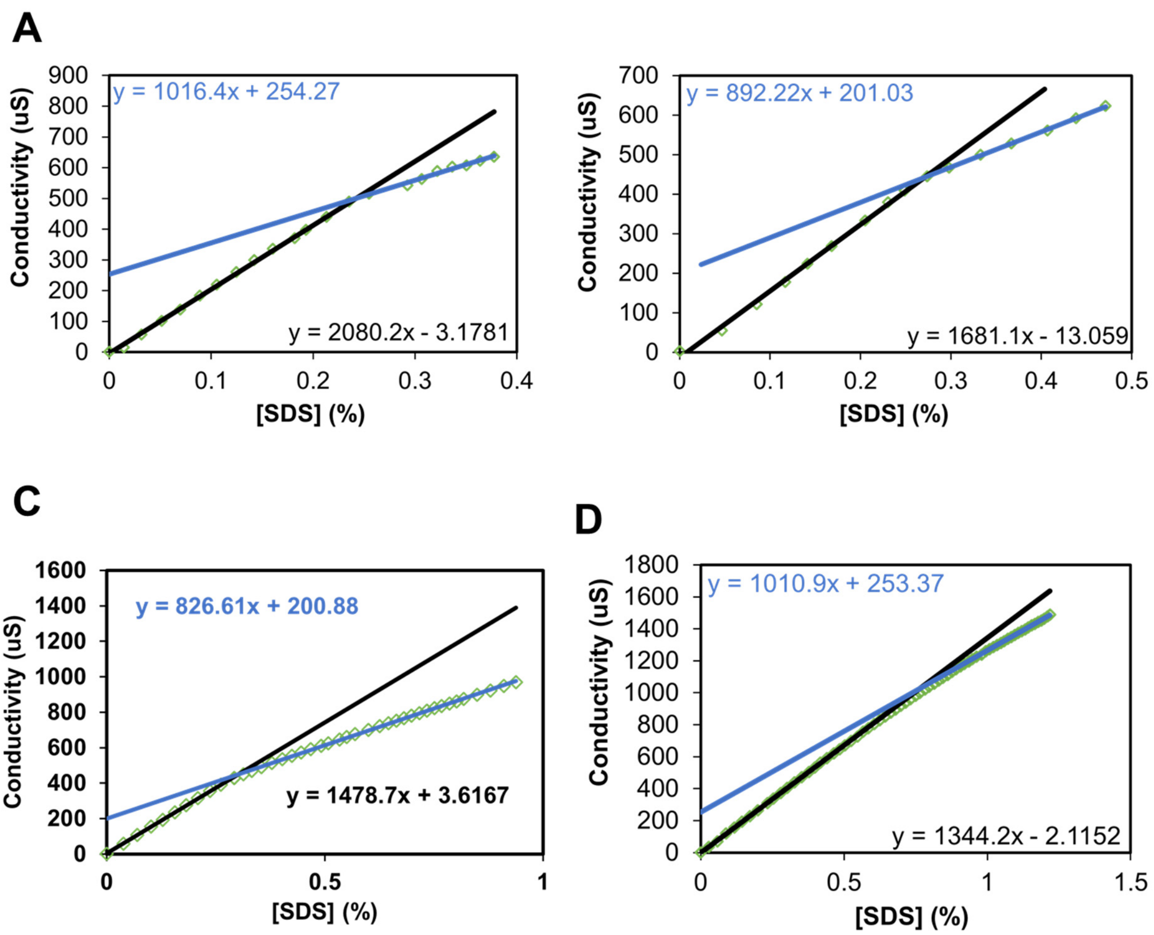

Figure S4. Plot of conductivity vs SDS concentration, whereby the breaks in the linear trendlines indicate the CMC. **A**) 0% methanol. **B**) 10% methanol. **C**) 20% methanol. **D**) 40% methanol. The intersection points correspond to SDS concentrations of 8.4 mM (pure water); 9.4 mM (10% methanol); 10.5 mM (20% methanol); and 26.5 mM (40% methanol).

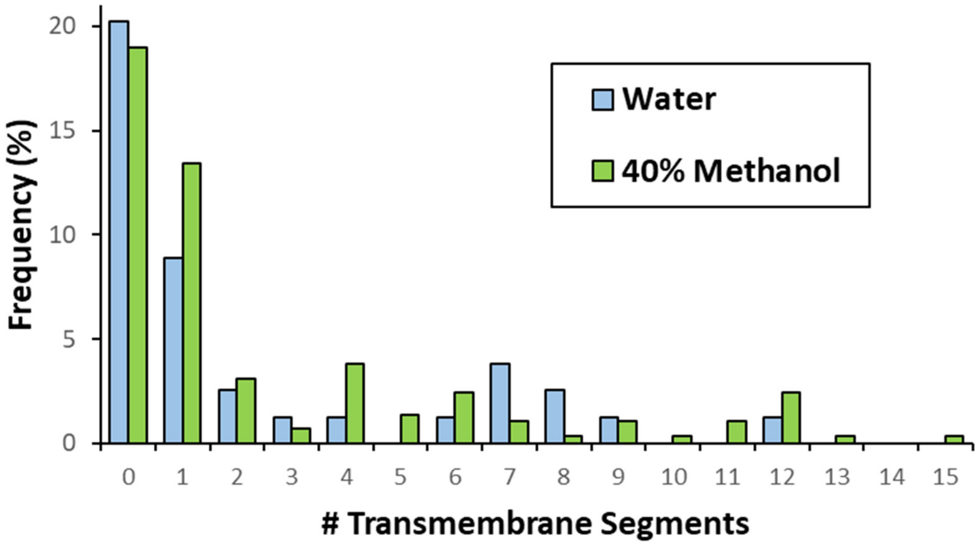

Figure S5. Histogram summarizing the number of transmembrane segments contained in membrane proteins determined to be enriched from the TME preparation with 40% methanol (green), or enriched in the water-containing TME preparation.

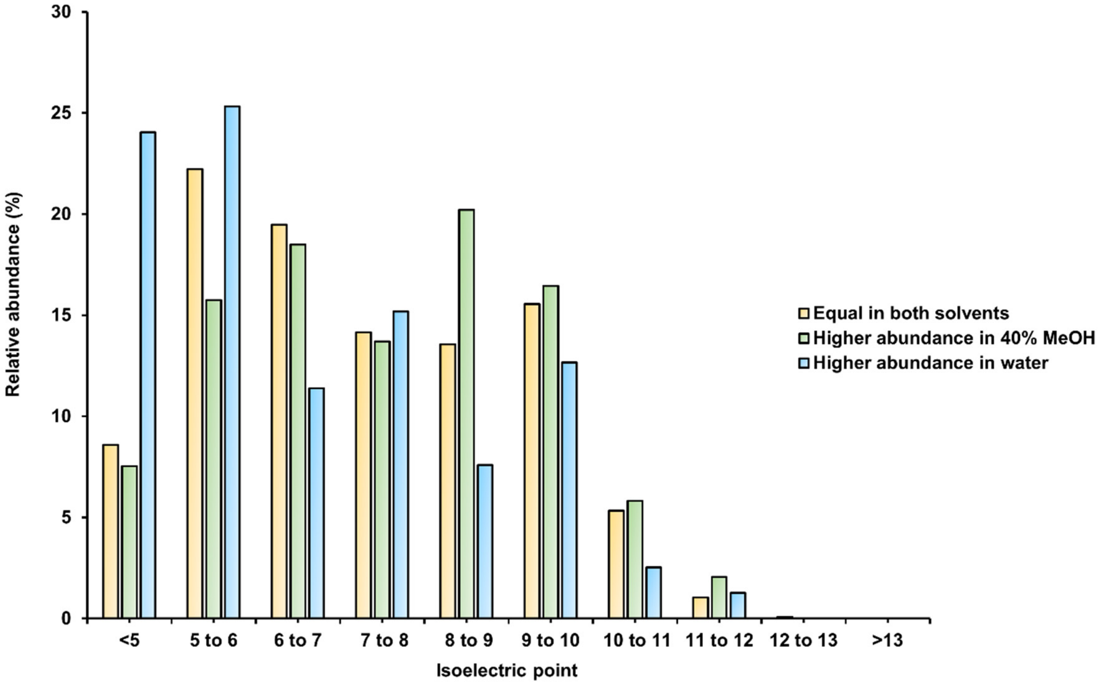

Figure S6. Histogram demonstrating the distribution of isoelectric point extracted in each condition. Proteins that are statistically enriched in the 40% MeOH preparation show a larger fraction of proteins with pI close to the TME operating buffer (pH 8.1).
